# Spatial Patterns of Decreased Cerebral Blood Flow and Functional Connectivity in Multiple System Atrophy (Cerebellar-Type): A Combined Arterial Spin Labeling Perfusion and Resting State Functional Magnetic Resonance Imaging Study

**DOI:** 10.3389/fnins.2019.00777

**Published:** 2019-07-31

**Authors:** Weimin Zheng, Shan Ren, Hao Zhang, Ming Liu, Qiuhuan Zhang, Zhigang Chen, Zhiqun Wang

**Affiliations:** ^1^Department of Radiology, Aerospace Center Hospital, Beijing, China; ^2^Department of Neurology, Dongfang Hospital, Beijing University of Chinese Medicine, Beijing, China; ^3^Department of Radiology, Dongfang Hospital, Beijing University of Chinese Medicine, Beijing, China

**Keywords:** multiple system atrophy, magnetic resonance imaging, regional cerebral blood flow, arterial spin labeling perfusion, functional connectivity

## Abstract

Multiple system atrophy (MSA) is a progressive neurodegenerative disease. However, little is known about the regional cerebral blood flow (rCBF) and functional connectivity changes in the disease. In this study, the magnetic resonance imaging (MRI) data including 24MSA-c-type patients and 20 healthy controls were collected by using voxel wise arterial spin labeling (ASL) perfusion analysis, several regions of the altered rCBF were identified in the MSA c-type patients. And then, the changes of the functional connectivities of identified rCBF regions were analyzed by using functional MRI (fMRI). Finally, rCBF value of cerebellum was extracted to differentiate the MSA c-type patients and controls. Compared with the controls, the MSA c-type patients showed distinct disruption of rCBF in the cerebellum. The disconnection of the identified cerebellar regions was revealed in several regions in the MSAc-type patients, including right middle frontal gyrus (MFG), right precuneus, left superior temporal gyrus (STG), right lingual gyrus, left postcentral gyrus (PoCG), right cerebellum 7b, right cerebellum 8, and left cerebellum 4,5. These regions were involved in the default mode network (DMN), sensorimotor network, visual associated cortices, and cerebellum. Using the rCBF value of vermis as biomarker, the two groups can be differentiated and reached a sensitivity of 95.8% and specificity of 100%. This is the first study to demonstrate the MSA-specific rCBF abnormalities using the ASL method, which are closely associated with several functional networks on resting state fMRI. The rCBF of vermis might be used as the potential imaging biomarker for the early diagnosis of MSA c-type.

## Introduction

Multiple system atrophy (MSA) is a progressive neurodegenerative disease characterized by parkinsonism with low dopamine response, cerebellar ataxia, and dysautonomic (Krismer and Wenning, 2017). The most important neuropathological feature is alpha synuclein-positive glial cytoplasmic inclusions (GCIs), resulting in the degeneration of neuron, especially in the striatum, cerebellum, and olivopontine structures (Brettschneider et al., 2017). Currently, it is mainly divided into MSA-p type with poor levodopa-responsive parkinsonian syndrome, and MSA-c type with cerebellar ataxia syndrome. In addition, the two types of MSA might be associated by autonomic dysfunction (Gilman et al., 2008).

Structurally, most MSA neuroimaging researches have been focused on the gray matter atrophy detected by the structural magnetic resonance imaging (MRI) (Matsusue et al., 2009; Deguchi et al., 2015; Chelban et al., 2018; Krismer et al., 2018; Dash et al., 2019). As the results, atrophy of the putamen, middle cerebellar peduncle (MCP), pons, or cerebellum on MRI reflected the pathological changes of MSA (Matsusue et al., 2009; Deguchi et al., 2015). In addition, several studies reported the slit-like hyperintense putaminal rim, MCP hyperintensities, and hot-cross bun sign (HCB) on MRI T2-weighted images (T2WI), which were helpful for the diagnosis of the disease (Chelban et al., 2018; Krismer et al., 2018; Dash et al., 2019). Previous MSA studies have focused on the volumetric and structural patterns of the disease. However, new functional techniques have provided deep understanding of the disease and improved diagnostic accuracy (Kim et al., 2017).

Functionally, resting-state functional MRI (rs-fMRI) has attracted increasing attention to be used to explore intrinsic brain activity and connectivity (Fox and Greicius, 2010; Iancheva et al., 2018; Kandilarova et al., 2018; Zhao et al., 2018). Several studies of MSA-p type found the disruption of the striatal-thalamo-cortical (STC) network, default mode network (DMN), visual associated cortices (Wang et al., 2017), as well as cerebello-thalamo-cortical (CTC) network (Yao et al., 2017). Furthermore, recent study of MSA-c type revealed the altered functional connectivity of cerebello-cortical circuit (Ren et al., 2019). These studies provided evidences for the hypothesis of “disconnection syndrome,” suggesting that the accumulation of alpha-synuclein GCIs of the MSA may destroy the specific networks including the STC, CTC, DMN, and so on (Yao et al., 2017; Rosskopf et al., 2018). However, the underlying physiological mechanism of brain function changes of MSA is not very clear.

Based on the previous study, underlying physiological mechanism of brain function was closely coupled with the cerebral perfusion, which can be detected by arterial spin labeling (ASL) analysis. As a non-invasive technique, ASL can provide quantitative information of regional cerebral blood flow (rCBF). In a previous study of healthy normal people, by combining ASL and fMRI analysis, the researchers found that functional brain hubs showed striking spatial correlation with rCBF (Liang et al., 2013). Recent experience in neurodegenerative disorders (Zhang, 2016) suggested that ASL may prove a useful and safe neuroimaging tool to detect the blood flow changes. At present, no investigation was performed to explore the changes of the rCBF in MSA and its affect on brain function changes. Therefore, we decide to combine the ASL method and rs fMRI simultaneously to explore the MSA patients, which may be helpful for deep understanding of the functional changes and underlying mechanism of rCBF dysfunction of MSA.

In this study, we focused on the MSA-c type. By combining the voxel wise ASL and resting state fMRI methods, we hypothesized that there might be decreased rCBF in some specific regions in MSA, which were closely associated with the functional changes. Furthermore, rCBF changes might be affected early in the disease course, we expected that ASL of specific regions can accurately describe and track disease progression, which can be applied as a valuable imaging biomarker for early diagnosis of MSA-c type.

## Materials and Methods

### Study Subjects

Twenty-four MSA patients and 20 controls were recruited at the clinic of Dongfang Hospital of Beijing University of Chinese Medicine. The two groups matched for age and gender. The diagnosis of MSA was according to the established international diagnostic criteria of probable MSA defined by the American Academy of Neurology and American Autonomic Society (Gilman et al., 2008). All subjects were evaluated by complete physical and neuropsychological examinations including Mini-Mental State Examination (MMSE), Montreal Cognitive Assessment (MoCA), and Unified Multiple System Atrophy Rating Scale (UMSARS). The clinical examinations were performed on the day before fMRI scanning.

The inclusion criteria for controls were as follows: (1) there were no neurological or psychiatric disorders including obsessive disorder, anxiety disorder, schizophrenia, depression, epilepsy, and so on; (2) there were lack of significant cognitive decline (MMSE score > 24); (3) there were no neurological deficiencies including visual or hearing loss; (4) there were no treatment with deep brain stimulation or operation; (5) there were no evidence of movement disorder, vascular brain lesions, brain tumor, and/or marked cortical and/or subcortical atrophy on MRI scan.

The exclusion criteria for the subjects were as follows: The subjects of hemorrhage, infarction, tumors, trauma, or severe white matter hyperintensity were excluded from the study. Clinical and demographic information of the subjects were shown in Table 1.

**TABLE 1:** Demographic and clinical characteristics of the participants.

**Characteristics**	**MSA-C (*n* = 24)**	**Control (*n* = 20)**	***p*-Value**
Age, years	57.29 ± 1.20	57.20 ± 1.11	0.715
Gender, male/female	14/10	7/13	0.383
Education, years	13.80 ± 0.48	13.75 ± 0.49	0.192
Disease duration years	4.27 ± 0.18	NA	
MMSE	27.20 ± 0.45	27.30 ± 0.42	0.374
MoCA	27.40 ± 0.29	28.10 ± 0.28	0.378
UMSARS-I, total	16.54 ± 1.17	NA	
UMSARS-II, total	16.17 ± 1.28	NA	
Over disability grade	2.49 ± 0.25	NA	

All subjects gave written informed consent in accordance with the Declaration of Helsinki. The protocol was approved by the Medical Research Ethical Committee of Dongfang Hospital of Beijing University of Chinese Medicine.

### Data Acquisition

Magnetic resonance imaging data acquisition was performed on a GE 3.0T Discovery 750 scanner. Foam padding and headphones were used to control head motion and scanner noise. The resting-state fMRI data was acquired by using the following parameters: repetition time (TR)/echo time (TE)/flip angle (FA) = 2,000 ms/30 ms/90°, field of view (FOV) = 24 × 24 cm^2^, resolution = 64 × 64 matrix, slices = 36, thickness = 3 mm, gap = 1 mm, voxel size = 3.75 × 3.75 × 3 mm^3^, and bandwidth = 2232 Hz/pixel. For volume calculation, high-resolution anatomical images were collected using a 3D brain volume (BRAVO) T1-weighted sequence with the following parameters: TR/TE/inversion time (TI)/FA = 8150 ms/3.17 ms/450 ms/12°, resolution = 256 × 256 matrix, slices = 188, thickness = 1 mm, voxel size = 1 × 1 × 1 mm^3^. 3D ASL data were acquired using the following parameters: TR/TE = 2.0 s/14 ms, Post label delay (PLD) = 2.0 s, FOV = 256 × 256 mm^2^, matrix size = 64 × 64, in plane resolution = 3 × 3 mm^2^, bandwidth = 2,232 Hz/px, phase partial Fourier = 6/8, EPI factor = 64. Twelve slices of 6 mm-thickness were acquired. All the participants were instructed to keep their eyes closed, move as little as possible, think of nothing in particular, and stay awake during the scans.

### rCBF Analysis

The images with differences in 3D-ASL were averaged, and the rCBF map was calculated with the weighted reference image of proton density (Xu et al., 2010). Image processing was performed using SPM12 software. Firstly, the rCBF images were normalized to the Montreal Neurological Institute (MNI) space. Then, to reduce the individual variance, the normalized rCBF images were standardized. Finally, each standardized rCBF map was spatially smoothed with a Gaussian kernel of 8 mm × 8 mm × 8 mm full-width at half maximum (FWHM).

### Functional Connectivity Analysis

Data Processing Assistant for Resting-State fMRI (DPARSF, Yan and Zang, 2010)^1^. The first 10 volumes for each participant were discarded to allow the signal to reach equilibrium and the participants to adapt to the scanning noise. The remaining volumes were corrected for the acquisition time delay between slices. Then, realignment was performed to correct the motion between time points. In resting state fMRI, a common finding is that many long-distance correlations are decreased by subject motion, whereas many short-distance correlations are increased. Therefore, we estimated subject head motion immediately after the fMRI scans to ensure that all participants’ data were within the defined motion thresholds (i.e., translational or rotational motion parameters less than 2 mm or 2°). To spatially normalize the fMRI data, the realigned volumes were spatially standardized into the MNI space using the EPI template. The functional images were resampled into a voxel size of 3 mm× 3 mm× 3 mm. Then, the functional images were smoothed with a Gaussian kernel of 4 mm FWHM. Finally, several nuisance covariates (six motion parameters, their first time derivations, white matter, and cerebrospinal fluid) were regressed out from the data. After preprocessing, time band-pass filtering (0.01–0.08 Hz) of the fMRI data reduced the effects of a low-frequency drift and high-frequency physiological noise, such as respiratory and cardiac rhythms.

To investigate change in network level function in MSA patients, we conducted a seed-based inter-regional correlation analysis. According to the rCBF result, there might be several regions identified as significant abnormalities in patients with MSA. We selected the regions presenting most significant difference between the two groups as cluster masks, and defined them as regions of interest for functional connectivity analysis. Correlation analysis between the time series of seed and the time series of the entire brain in a voxel-wise way was performed. The value of *z* is obtained using Fisher’s r-to-z transformation to improve the Gaussianity of its distribution.

### Voxel-Based Morphology (VBM) Analysis

To eliminate the influences of brain atrophy on the resting state functional connectivity, we took voxel wise gray matter (GM) and white matter (WM) volumes as covariates during the rCBF analysis. First, each subject’s GM and WM volume map was estimated from the normalized T1WI images by VBM method (VBM8 toolbox). Then, a two-sample was performed between the MSA and controlgroup, which showed brain atrophy in the MSA patients.

### Statistical Analyses

For between-group comparisons (rCBF differences between the MSA patients and the controls), we use voxel-wise Family-wise error (FWE) rate correction (threshold at voxel level *p* < 0.05) to conduct the multiple comparison corrections, with age, gender, education levels, GM and WM volumes as covariates using Statistical Parametric Mapping software package (SPM12)^2^.

Then, the regions that were significantly changed in terms of the rCBF were selected as areas of interest (ROIs). The whole brain functional connectivity of the ROIs was computed and independent-samples *t*-test analyses were performed to investigate the functional connectivity differences between MSA patients and controls. The significance threshold was set to voxel level of *p* < 0.05 (FWE correction).

To explore the associations of the clinical variables with the rCBF and connectivity in the MSA patients, a partial correlation analysis was performed with age, gender and education levels being used as nuisance covariates (*P* < 0.05).

Finally, we use receiver operating characteristic (ROC) analysis with SPSS 20.0 to obtain a sensitivity and specificity imaging biomarker for MSA diagnosis.

## Results

### Demographic and Neuropsychological Tests

Demographic and clinical characteristics were described in Table 1. No significant differences of gender, age, education, MMSE and MoCA scores were found between the MSA-c type and control groups. However, The UMSARS scores were described in the MSA-c type patients which referred to the severity of the disease.

### rCBF Changes Between the MSA-C Type and Controls in the Resting State

**FIGURE 1:**
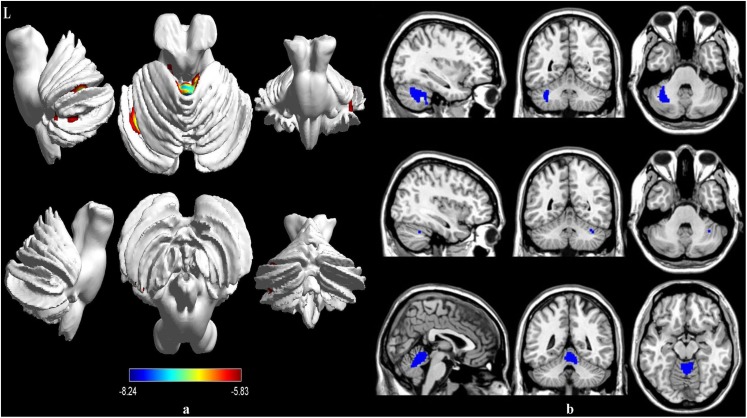
Voxel-wise percentage CBF changes in patients with MSA compared with healthy controls. Decreased rCBF in MSA patients compared to healthy controls were mainly located in the left cerebellum 6, right cerebellum crus 1 and vermis 4,5 (with FEW-corrected *P* < 0.05). FEW = Family-wise error.

Compared to the healthy controls, the patients with MSA-c type showed significantly decreased rCBF in the left cerebellum 6, right cerebellum crus 1 and vermis 4,5. The peak voxels within those significantly different clusters were shown in Figure 1 and Table 2.

**TABLE 2:** Regions of significant different rCBF between MSA-c type and controls.

**ROIs**	**Cluster voxels**	**MNI coordinates**			**Maximum *Z***
		***x***	***y***	***z***	
Cerebelum_6_L	481	−32	−64	−28	−7.0775
Cerebelum_Crus1_R	13	36	−52	−32	−5.8305
Vermis_4_5	716	4	−50	−12	−8.237

*ROIs, regions of interest.*

### Functional Connectivity Between MSA-c Type Group and Controls

**FIGURE 2:**
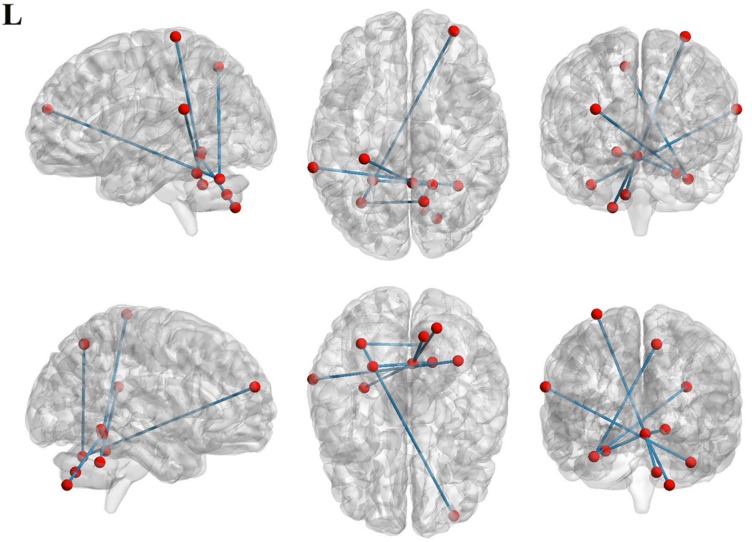
Functional connectivities alterations between the three selected regions and whole brain other regions in the MSA patients relative to controls.

To investigate functional connectivity alterations in the MSA-c type patients, seed-based interregional correlation analysis was performed. We selected the three regions of bilateral cerebellum, which were significantly changed in rCBF as seeds. In the Figure 2 and Table 3, MSA-c type patients exhibited decreased connectivities between the three selected regions and several other regions, including the right middle frontal gyrus (SFG), right precuneus, left superior temporal gyrus (STG), right lingual gyrus, left postcentral gyrus (PoCG), right cerebellum 7b, right cerebellum 8, and left cerebellum 4,5.

**TABLE 3:** Regions of significant different connectivity between MSA-c type and controls.

**ROIs**	**Brain regions**	**Cluster voxels**	**MNI coordinates (mm)**			**Maximum *Z***
			***x***	***y***	***z***	
Cerebelum_6_L	Frontal_Mid_R	6	33	57	21	−6.2823
	Precuneus_R	5	12	−63	51	−6.6963
Cerebelum_Crus1_R	Temporal_Sup_L	8	−66	−39	21	−6.2662
Vermis_4_5	Cerebelum_7b_R	3	21	−75	−48	−6.9004
	Cerebelum_8_R	1	12	−69	−39	−5.8121
	Cerebelum_4_5_L	1	−24	−48	−24	−5.9745
	Lingual_R	1	18	−51	−9	−5.9732
	Postcentral_L	2	−30	−33	72	−6.559

*ROIs, regions of interest; MFG, middle frontal gyrus; STG, superior temporal gyrus.*

### Correlation Between rCBF, Functional Connectivity, and Clinical Performances in the MSA-c Type Group

In the MSA-c type group, we didn’t find significant correlations between the UMSARS scores and rCBF as well as functional connectivity changes.

### The rCBF Analysis of Vermis as Biomarker

**FIGURE 3:**
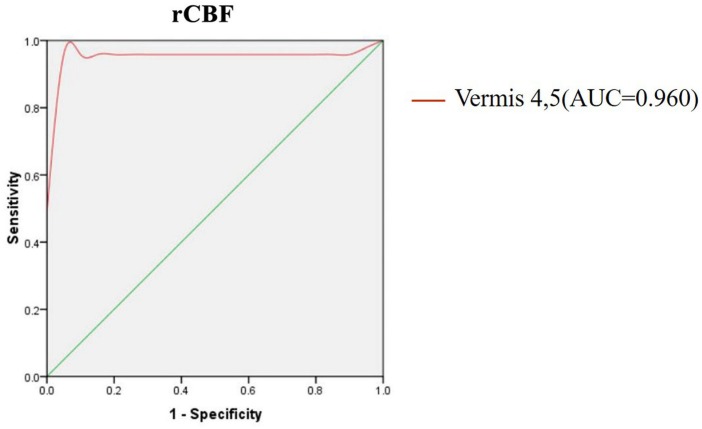
ROC curve for CBF in vermis of patients with MSA and healthy controls. An optimal CBF cutoff value was determined at a sensitivity of 95.8% and specificity of 100%. The area under the curve (AUC) for the ROC was 0.960 (95% confidence intervals from 0.884 to 1.037).

As shown above, the vermis was the region with the most significant rCBF differences between the two groups, which raised a possibility that the rCBF values for the vermis might serve as markers to differentiate the MSA patients from healthy controls. To explore the possibility, we calculated the rCBF value of each subject within vermis 4, 5 and performed ROC analysis to differentiate the MSA patients and the healthy controls. Figure 3 showed the ROC analysis results. Using the cut-off rCBF value of 0.958, we could differentiate the two groups with a sensitivity of 95.8% and specificity of 100%. The area under the curve (AUC) of the ROC was 0.960 (95% confidence intervals from 0.884 to 1.037).

## Discussion

By performing ASL analysis, we observed significant decreased rCBF values of several cerebellum regions in MSA-c type patients. By using the altered rCBF regions as seeds, we found functional disconnectivities between the selected cerebellum regions and several other regions including the right MFG, right precuneus, left STG, right lingual, left PoCG, right cerebellum 7b, right cerebellum 8, and left cerebellum 4,5. Importantly, these regions were involved in several specific networks including default mode network, sensorimotor network, visual associated cortices and cerebellum. Finally, we found the rCBF value of vermis could be used as a sensitive biomarker to differentiate MSA and controls. These findings might be helpful for us to understand the neural pathophysiology mechanisms of MSA.

### rCBF Changes Between the MSA Patients and Controls in the Resting State

Decreased rCBF values were revealed in several cerebellum regions in MSA-c type patients, suggesting the impaired cerebral perfusion in the disease. Typical histopathological findings of MSA-c type have been observed predominantly in cerebellum. Structurally, recent study found the disrupted structural atrophy in the cerebellum of the MSA-c type, presenting gray matter loss and white matter degeneration (Dash et al., 2019). Functionally, previous study confirmed the impaired intrinsic brain activity limited in the cerebellum in the MSA-c type patients (Ren et al., 2019). However, no investigation was performed to explore the changes of the cerebral blood flow in MSA. In this study, we firstly identified the regions of the decreased perfusion, which is meaningful for understanding the underlying mechanism of the impaired structure and function of the MSA-c type. According to the neurovascular coupling hypothesis (Kuschinsky, 1991; Liang et al., 2013; Venkat et al., 2016), rCBF changes might contribute to altered structural and neuronal activity in the MSA-c type patients. Specifically, the disrupted cerebral perfusion of MSA-c type might cause impaired vascular clearance ability, which promoted accumulation of alpha synuclein-positive GCIs, and finally exerted neurotoxic effects on the brain leading to neurodegeneration. Therefore, we speculated that the vascular dysfunction of cerebellum might result in the abnormalities of the structure and function in the MSA c-type patients, which was consistent with the clinical main symptom of cerebellar ataxia of the disease.

### Functional Connectivity Changes Between the MSA-c Type Patients and Controls in the Resting State

Based on the altered rCBF regions, we revealed the disrupted functional connectivity pattern in MSA-c type, which exhibited functional disconnection between the cerebellums and several brain regions, involving the DMN, sensorimotor network (SMN), and cerebellar network.

We found the disrupted connectivity between the cerebellum and the several DMN regions (right MFG, right precuneus, left STG) in the MSA-c type patients. As we known, these regions were functionally connected with each other and constituted the primary hub of the DMN during resting state (Buckner et al., 2008; Ward et al., 2014). The DMN was a functional-anatomic network implicated in memory, self-reflection, and stream-of-consciousness processing (Greicius et al., 2004; Buckner et al., 2008). The disruption of DMN was consistently demonstrated in Alzheimer’s disease (AD) by many resting state fMRI studies, which contributed to the memory deficit of AD (Zheng et al., 2017; Liu et al., 2018). However, with the gradually deep understanding of the DMN, researchers found the changes of DMN in several other neurological disorders, including depression (Sheline et al., 2010), autism spectrum disorders (Assaf et al., 2010), schizophrenia (Rotarska-Jagiela et al., 2010), as well as in MSA-p type (Franciotti et al., 2015). These reported findings indicated that the DMN regions might be responsible for multiple functions beyond the memory, including emotion, action, mental imaginary, cognition, interception, and perception. For example, the frontal gyrus was involved in motor preparation and endogenous motor plans (D’Esposito et al., 2000; Koechlin et al., 2000). Therefore, the DMN disruption in the MSA-c type may contribute to the impairment of the multiple function. This result was consistent with the previous MSA studies, which also reported the disruption of DMN in the MSA-c type patients (You et al., 2011; Yao et al., 2017; Rosskopf et al., 2018; Ren et al., 2019).

Besides, we noticed decreased connectivity between the cerebellum and the region of PoCG, which was involved in the SMN. This network was constituted by primary sensory and motor cortices, indicating the important role of the related function (Beckmann et al., 2005; De Luca et al., 2006). By exploring the intrinsic brain activity, a previous study revealed altered regional homogeneity (ReHo) in sensorimotor related areas in MSA patients, suggesting the sensorimotor circuit dysfunction of the disease (Chelban et al., 2018). Furthermore, pathological changes were involved in primary sensorimotor cortices, premotor cortices, and supplementary motor areas (SMA) in MSA patients (Su et al., 2001; Yoshida, 2007).Together, we speculated that along with sensorimotor impairment in MSA-c type, the functions of the sensorimotor system might be degenerated.

We found decreased connectivity between the cerebellum and the right lingual gyrus in MSA c-type patients. The lingual gyrus is a structure in the visual cortex that plays an important role in processing vision. In a previous study, impaired visual memory was associated with the gyrus or disconnections between the gyrus and other brain structures (Kozlovskiy et al., 2014). Recent study confirmed the abnormality of intrinsic brain activities in visual associative cortices in MSA p type patients (Wang et al., 2017). In this study, we revealed the disconnection of lingual gyrus and the cerebellum, which provided the new evidence for the disconnection pattern of the MSA-c type patients. Further study needs to be investigated in the future.

Finally, we also detected the decreased connectivity among the several different cerebellum regions including right cerebellum 7b, right cerebellum 8, and left cerebellum 4,5. To our knowledge of cerebello-cortical circuit: The afferent fibers of cerebellum mainly come from the opposite cerebellopontine nucleus and the inferior olivary nucleus, passing through the middle and lower cerebellar peduncles to the new cerebellum, and then, the cerebellar cortex sends the efferent fibers to dentate nuclei and forms the main body of the superior cerebellar peduncles, which project into the contralateral thalamus and cerebral cortex. From the view of the process of the cerebello-cortical circuit, many cerebellum regions played an important role in the network, which was responsible for the balance, planning, and coordination of motor functions. In this study, the disconnection within the several cerebellum regions was consistent with the pathology of the MSA-c type, which emphasized on the cerebellum atrophy and dysfunction (Matsusue et al., 2009; Deguchi et al., 2015). In addition, these results were matched well with several previous resting state fMRI studies (You et al., 2011; Yao et al., 2017; Rosskopf et al., 2018; Ren et al., 2019).

### The rCBF Analysis of Vermis as Biomarker

In the current study, by using the rCBF of the vermis as the biomarker, we could differentiate the two groups at the cut-off value of 0.958 yielding a sensitivity of 95.8%, specificity of 100%, and the AUC value of 0.960. It was an interesting result which could be used as valuable imaging biomarkers for the early diagnosis of MSA-c type.

In the previous study, most researchers used structure changes as biomarker to differentiate the MSA-c type and controls. For example, a previous study used the “hot cross bun” sign as biomarker to diagnose MSA-c type, yielding a high specificity of 97%, but its sensitivity was only 50% (Yekhlef et al., 2000). While another study used the brainstem atrophy as biomarker to differentiate the two groups, which reached the sensitivity of 100% and specificity of 82% (Chelban et al., 2018). In our study, we firstly employed the ASL on MSA patients and extracted the rCBF of vermis as biomarker to diagnose the disease, which reached high specificity and sensitivity simultaneously.

### Future Considerations

There are still some issues to be addressed. First, in the current study, we mainly focused on the cerebral perfusion and functional connectivity. Further studies using multi-mode methods including resting state and task related fMRI, diffusion, perfusion, and spectroscopy analysis would be more helpful for deep understanding the mechanism of MSA. Second, recent studies have paid more attention to MSA p-type. In the future, exploring MSA different subtype (parkinsonian and cerebellar variants) would provide valuable biomarkers for the early differential diagnosis of the disease. Finally, we didn’t detect the correlation between the parameter changes and the clinical performances; in the future, a large sample of fMRI data needs to be performed to test the current findings and a longitudinal design would be useful to elucidate the progressive functional changes of the MSA patients and its relationship with clinical performances.

## Conclusion

In conclusion, we identified the regions of significantly decreased rCBF in the MSA patients, which were mainly located in cerebellum. In addition, we found the disrupted functional connectivity of the selected cerebellum regions in the MSA patients, which were involved in different functional brain networks such as DMN, SMN, visual associate cortices and cerebellar network. These findings added the new evidence for the disrupted rCBF and disconnection syndrome of MSA, which might provide the potential biomarker for detecting early MSA in the future.

## Data Availability

The datasets analyzed in this manuscript are not publicly available. Requests to access the datasets should be directed to wangzhiqun@126.com.

## Ethics Statement

The studies involving human participants were reviewed and approved by Medical Research Ethical Committee of Dongfang Hospital of Beijing University of Chinese Medicine. The patients/participants provided their written informed consent to participate in this study.

## Author Contributions

ZC and ZW carried out the research project and conceived the study. ML organized the study. HZ and QZ executed the results. WZ carried out the statistical analysis and designed the study. HZ executed the results. ZW reviewed and critiqued the manuscript. WZ and SR wrote the first draft of the manuscript.

## Conflict of Interest Statement

The authors declare that the research was conducted in the absence of any commercial or financial relationships that could be construed as a potential conflict of interest.

## Footnotes

1 http://rfmri.org/DPARSF

2 http://www.fil.ion.ucl.ac.uk/spm
